# Self-Rated Oral Health as a Valid Measure of Oral Health Status in Adults Living in Rural Australia

**DOI:** 10.3390/healthcare11121721

**Published:** 2023-06-12

**Authors:** Claudia Atala-Acevedo, Roisin McGrath, Kristen Glenister, Daniel Capurro, Lisa Bourke, David Simmons, Mike Morgan, Rodrigo Mariño

**Affiliations:** 1 Melbourne Dental School, The University of Melbourne, Melbourne, VIC 3010, Australia; 2 Faculty of Dentistry, Universidad de La Frontera, Temuco 4811230, Chile; 3 Department of Rural Health, The University of Melbourne, Shepparton, VIC 3630, Australia; 4 School of Computing and Information Systems, The University of Melbourne, Melbourne, VIC 3010, Australia; 5 Centre for the Digital Transformation of Health, Melbourne, VIC 3010, Australia; 6 Macarthur Clinical School, Western Sydney University, Sydney, NSW 2000, Australia

**Keywords:** self-rated oral health, oral health status, adults, rural Australia

## Abstract

It is unclear how well self-rated oral health (SROH) reflects actual oral health status in the rural Australian population. Therefore, this study aimed to compare the clinically assessed oral health status and SROH of adults living in rural Australia. The data were from 574 participants who took part in the Crossroads II cross-sectional study. Three trained and calibrated dentists evaluated the oral health status of participants based on WHO criteria. SROH was assessed with the question ‘Overall, how would you rate the health of your teeth and gums?’, with a score ranging from excellent = 5 to poor = 1. A logistic regression analysis (LRA) was performed, allowing us to assess factors associated with SROH. The mean age of participants was 59.2 years (SD 16.3), and 55.3% were female. The key results from the LRA show poorer SROH in those with more missing teeth (OR = 1.05; 95% CI; 1.01–1.08), more decayed teeth (OR = 1.28; 95% CI: 1.11–1.46), and more significant clinical attachment loss of periodontal tissue (6mm or more) (OR = 2.63; 95% CI: 1.29–5.38). This study found an association between negative SROH and clinical indicators used to measure poor oral health status, suggesting that self-rated oral health is an indicator of oral health status. When planning dental healthcare programs, self-reported oral health should be considered a proxy measure for oral health status.

## 1. Introduction

Oral health is a multidimensional construct wherein physical, psychological, and social aspects contribute to a person’s perceived oral health status and general health and wellbeing [1]. A healthy mouth is essential for eating, speaking, smiling and socialising [2].

People in rural areas of Australia experience poorer oral health than individuals living in major cities [2]. This is, in part, related to health literacy, social determinants, differences in health behaviours, and the barriers to accessing dental care services in rural areas, such as distance, high costs and long waiting lists for public dental care [3]. Therefore, it is important to increase the use of oral health assessments, clinical and self-reported, in people living in rural areas to establish a profile of dental care needs and plan oral health services and prevention programs.

When assessing oral health status, it is common practice for oral health professionals to focus on clinical measures and not consider the patient’s perception of their oral health [4]. Nowadays, the perception of oral health and self-rated oral health (SROH) is becoming more important because it has been linked with the quality of dental care and helps us to understand the impact of oral health status on an individual’s quality of life [5,6,7]. As SROH provides insight into individuals’ oral health status, it can provide a more cost-effective approach for large-scale cross-sectional population studies.

Studies have found an association between self-reported oral health and clinical oral health assessment, for example, a poor SROH and few natural teeth, a high number of decayed teeth, the presence of gingival bleeding, toothache and speech and chewing problems [6,7,8]. Poor SROH has also been reported as a predictor of tooth loss between five and ten years into the future [9]. Furthermore, ethnicity, socioeconomic status, dental visiting patterns and oral care habits greatly influence people’s perceptions of their oral health [8,10].

Although there is some information available at the national level and for each state of Australia about SROH and oral health profiles from the National Study of Adult Oral Health (NSAOH) [11], there is a scarcity of research exploring the association between oral health status and SROH in rural Australia. SROH in the rural population may differ from that in urban areas; therefore, research on the perception of oral health in the Australian rural population could provide valuable information for the development of more effective oral health promotion strategies. While some studies have shown SROH as a valid measure in evaluating the oral health status, it has not yet been investigated in the rural Australian population. So, the question that needs to be answered is the following: Can we use SROH as a valid measure of oral health status in the rural Australian population? If yes, a SROH tool could be used to screen these populations efficiently and economically to identify unmet oral healthcare needs for planning dental services. Thus, this study compares clinically assessed oral health status and self-rated oral health in adults living in rural Victoria, Australia.

## 2. Materials and Methods

### 2.1. Study Design and Participants

This study was a secondary analysis of data from Crossroads II, a cross-sectional study of randomly selected households in rural Victoria, a south-eastern state in Australia [12]. The data were collected between 2016 and 2018 through face-to-face surveys and included 2680 participants older than 16 years. A subgroup of this sample (aged ≥ 18 years, non-pregnant people, *n* = 574) also underwent an oral health examination (dental sub-study). Based on previously published recommendations [13,14], this sample size (*n* = 574) was deemed large enough to provide statistical power (β = 0.80) to perform multiple logistic regression analyses (up to ten covariables) and to identify factors associated with SROH.

### 2.2. Self-Rated Health and Self-Rated Oral Health

Self-rated general health was assessed based on a single question with five response options (excellent, very good, good, fair and poor): ‘In general, would you say that your health is?’. The self-rated oral health question asked: ‘Overall, how would you rate the health of your teeth and gums?’. The same five response options were provided (excellent, very good, good, fair and poor). For the present study, responses were dichotomised into ‘positive’ (from excellent to good) and ‘negative’ (fair–poor).

Five questions about the self-reported presence or absence of oral health conditions were also included in the survey asking about toothache and mouth pain, decayed teeth, gingival bleeding, tooth mobility and the frequency of pain. Regarding the frequency of toothache, the question was: ‘In the last 12 months, how often have you had painful aching in your mouth?’. Four response options (never, hardly ever, occasional and fairly often) were provided. This variable was regrouped to make a clearer comparison in the logistic regression, coded as: ‘never and hardly ever’ and ‘occasional and fairly often’. Participants were also asked ‘Do you have your own teeth?’ with three response options (yes, no, partial). Appendix A shows the self-rated oral health questions selected.

### 2.3. Oral Health Assessment

Oral health status was assessed according to the World Health Organization (WHO) criteria for Oral Health Surveys [15]. The clinical examinations were performed by three previously trained and calibrated dentists. Written consent was obtained from each participant [12]. Their dental caries experience was calculated using the Decayed, Missing and Filled (DMFT) Index. The DMFT score is calculated as the sum of the number of decayed teeth, teeth missing due to caries and filled permanent teeth. Additionally, for dentate participants, unmet restorative treatment need was calculated by dividing the sum of decayed teeth by the sum of decayed and filled teeth [D/(D + F)] [16].

Based on the number of teeth present at the dental exam, the participants were divided into three categories: ‘edentulous’ (absence of all-natural teeth); ‘inadequate dentition’ (those with between 1 to 20 natural teeth); and ‘adequate dentition’ (≥21 teeth present) [17]. The presence of oral mucosal lesions and the wearing of dentures (upper/lower) are also described according to the WHO guidelines [15].

For periodontal status, the Community Periodontal Index (CPI) and clinical attachment loss (CAL) were used. The Community Periodontal Index was modified to have two indicators, gingival bleeding (0 = absence of bleeding, 1 = presence of bleeding) and periodontal pockets (0 = absence of a condition, 1 = pocket 4–5 mm, 2 = pocket ≥ 6 mm). Clinical attachment loss (the progressive loss of gum tissue due periodontitis) was measured by probing six sites per index tooth. The codes for loss of clinical attachment are 0 = 0–3 mm; 1 = 4–5 mm; 2 = 6 mm or more.

### 2.4. Sociodemographic Variables

The sociodemographic variables included were age, sex; level of formal education (with the categories including ‘some secondary’, ‘secondary complete’, ‘trades’ and ‘tertiary’); and the locality of residence, which was divided into the ‘regional centre’ (Shepparton/Mooroopna) and the ‘shire capitals’ (Benalla, Cobram and Seymour). Dental service utilisation was categorised as ‘12 months or less’, ‘between one and two years’ and ‘longer than 2 years’.

### 2.5. Statistical Analysis

Descriptive statistics were used for sociodemographic factors and oral health conditions and diseases. The bivariate association between the dental clinical variables and self-rated oral health was determined using the chi-square test. For the continuous response variables (e.g., the DMFT score), ANOVAs were used. For all cases, a level of significance of *p* < 0.05 was considered statically significant. The relationship between clinical oral health variables and self-rated oral health variables was assessed using a logistic regression analysis. The model employed a backward selection method, with case-wise deletion of cases with missing values. The predictors were included in the model based on the statistical significance of *p* < 0.20 from the bivariate analysis. Data analyses were conducted using IBM-SPSS Statistics (Version 27.0).

## 3. Results

A total of 574 adults participated in the Crossroads II dental sub-study, of whom 55.3% were female. The age of the participants had a mean of 59.2 years (SD 16.3) and ranged from 20 to 92 years. Almost half (48.6%) of participants were from the regional centre (Shepparton/Mooroopna), and 51.3% were from the shire capitals (Benalla/Cobram/Seymour). For nearly half (47.9%) of participants, their highest level of education was secondary school. Half of the study population (50.9%) reported having visited the dentist in the last 12 months or less, and 26.9% answered that they had not visited the dentist for more than two years. The sociodemographic characteristics of the sample are available in Table 1.

### 3.1. Self-Rated General and Oral Health Status

A third of the study sample (33.1%) self-rated their general health as ‘fair’, and 13.9% perceived their health to be ‘poor’. Participants’ self-assessment of their oral health was positive (75.1%). However, a quarter of participants’ SROH was ‘fair’ (14.4%) or ‘poor’ (10.5%). In reporting the presence of oral health conditions, 22.6% indicated they had gingival bleeding, 27.4% reported dental caries, 8.5% had a loose tooth, and 10.1% indicated they were experiencing tooth pain. When asked about their frequency of toothache, 46.7% of the participants answered that they ‘never felt pain’, 27.0% ‘hardly ever’, and 21.0% ‘occasionally’ felt pain. In response to the question asking if they had their own teeth, 10.2% (*n* = 56) answered ‘no’ (edentulous), and 25.0% (*n* = 137) reported ‘partial’ dentition.

### 3.2. Overall Oral Health Status

The majority of participants had more than 21 teeth (67.1%); however, a quarter (24.4%) had inadequate dentition (fewer than 20 teeth). Only 8.5% of participants in this study were edentulous. Around a third of participants (30.7%) wore an upper denture; 21.4% (*n* = 123) used a full upper denture, and 9.2% used a partial denture. Only 16% of the participants wore a lower denture. Most of the participants included in this study (83.4%) had no oral mucosal lesions. Of those with oral mucosal lesions, most cases were leucoplakia (16.6%; *n* = 76).

Dental caries

Among dentate participants (*n* = 525), the overall caries prevalence (presence of dental caries) was 55.2%. The mean DMFT score was 18.7 (SD 8.4). For DMFT components, participants had a mean of 1.3 (SD 1.9) decayed teeth (DT) and a mean of 6.4 (SD 5.2) teeth with fillings (FT). Regarding the unmet restorative needs, it was found that 39.6% (95% confidence interval) were unmet.

Level of education was significantly associated with the DMFT score (*p* < 0.001). Participants with a lower educational level (some secondary education) presented a higher mean dental caries experience (23.2, SD 6.5) compared to the groups who had completed tertiary education (14.4, SD 9.9). Additionally, statistically significant associations were found between dental caries experience and the locality of residence, with people living in the shire capitals having a higher mean of DMFT (20.0, SD 8.3) than the participants who live in the regional centre (17.5, SD 8.3) (*p* < 0.001). There were no significant differences in dental caries experience dependent upon respondents’ sex or timing of last visit to the dentist.

Periodontal status

Almost one-fifth (19.7%) of the participants had periodontal pockets between 4 and 5 mm in depth, with 7.8% (*n* = 45) having pockets measuring 6mm or more. Periodontal pockets greater than 4mm in depth were found more frequently in women (17.2%) than in men (10.3%) and among those with some secondary education (35.4%) than those who had completed tertiary education (17.7%). More pathological pockets were found in participants from the shire capitals (61.1%) than in the regional centre (38.9%).

Clinical attachment loss (CAL) between 4 and 5 mm was found in 33.2% (*n* = 191) of participants, and 6mm and over CAL in 17.9% (*n* = 103). Close to half (49.0%) of the dentate participants had no clinical attachment loss.

### 3.3. Association between Self-Rated Oral Health and Clinical Oral Health Status

In the bivariate analysis, self-rated oral health was significantly associated with the timing of last dental visit (*p* = 0.007) and self-reported general health (*p* < 0.001). Participants visiting the dentist ‘longer than two years’ ago were more likely to rate their oral health negatively (OR = 2.19; 95% CI: 1.45–3.32). Participants who reported their general health as ‘poor’ or ‘fair’ were also more likely to have less favourable SROH (OR = 3.58; 95% CI: 2.24–5.71).

Poorer SROH was associated with a greater number of Decayed, Filled and Missing teeth (DMFT score). Similarly, poorer SROH was associated with more severe periodontitis. Those with CAL ≥ 6 mm also reported poorer SROH than those with no or less than 6 mm of CAL. The details of the bivariate analysis are provided in Table 2.

Ten covariates were included in the model: level of education, last dental visit, self-rated general health, number of teeth, unmet restorative needs, decayed teeth, filled teeth, clinical attachment loss, toothache and the frequency of toothache. The final model included seven statistically significant factors associated with negative self-rated oral health (χ^2^ (7) = 74.61; *p* < 0.001).

Of the sociodemographic variables included in the model, having a last visit to the dentist longer than two years ago increased the odds of having a negative perspective of oral health (OR = 2.14; 95% CI: 1.17–3.93). People who perceived their general health to be poorer were also more likely to have negative self-rated oral health (OR = 2.32; 95% CI: 1.26–4.29). Of the dental clinical indicators included, having more missing teeth increased the odds of having negative self-reported oral health (OR = 1.05; 95% CI; 1.01–1.08). Those with decayed teeth were 1.28 times more likely to have negative self-rated oral health (OR = 1.28; 95% CI: 1.11–1.46). Similarly, participants with CAL ≥ 6 mm were more likely to have negative self-rated oral health (OR = 2.63; 95% CI: 1.29–5.38). Those who reported that they experienced toothache had 2.33 times greater odds of rating their oral health negatively compared to those who did not experience toothache (OR = 2.33; 95% CI: 0.97–5.56). Of those who experienced toothache, those who had more frequently experienced toothache (occasional or often) were 2.56 times more likely to rate their oral health as poor or fair than those who never experienced toothache (OR = 2.56; 95% CI: 1.35–4.84). The variance for predicting negative self-rated oral health using the final model was 27.7% (Nagelkerke r^2^ = 0.277) (see Table 3 for the final model).

## 4. Discussion

In this study, around a quarter (24.9%) of participants rated their oral health negatively. This was related to a high number of missing and decayed teeth and more severe clinical attachment loss (6mm or more). Additionally, experiencing toothache and timing of last dental visit were significantly associated with perceptions of oral health status.

An interesting point to highlight is that in comparison with the Crossroads I study (2000–2003) [18], wherein participants were asked to self-report the presence of natural teeth, the number of edentulous people decreased from 14.5% to 10.2%, which indicates a decrease in the prevalence of edentulism in this rural area of Victoria in the last eighteen years.

Half of the adult dentate population had caries (55.2%), and CAL greater than 4 mm (51.1%). While the findings from this study are consistent with several previous studies conducted in rural populations [2,19,20], there are some significant differences in the profile of oral health status between Australian rural and urban populations. For example, the DMFT Index in this study (18.7) was considerably higher than that in the urban population in Victoria (10.8) [2]. Although the studies are not directly comparable due to their methodological differences, this finding indicates a higher caries prevalence in this rural population. The number of decayed teeth was associated with poor SROH. This finding is consistent with similar studies [7,8,21,22], where participants with more dental caries (DMFT) had poorer SROH. This supports the idea of incorporating SROH both in large-population studies and dental practices since SROH reflects aspects not explained by the oral clinical assessment.

The high prevalence of periodontitis found is a cause for concern. In the present study, nearly twice as many adults with periodontitis (47.8%) were found compared to the proportion of adults with periodontitis across the state of Victoria (27.7%) [2]. Furthermore, an association was found between periodontal status and poor SROH, which is in line with the findings of other studies [7,8,10]. The CAL measurement, in addition to allowing us to evaluate the severity and amount of periodontal tissue lost, can also indicate the current or past presence of periodontitis [23]. People with more severe CAL are more likely to rate their oral health as poor (4–5 mm OR = 1.90; 95% CI: 1.02–3.54 vs. 6 mm or more OR = 2.63; 95% CI: 1.29–5.38), which could indicate a level of oral health awareness, and problems with functioning and mouth discomfort. Periodontitis is a chronic and silent condition. Often, the first sign that people pay attention to is dental mobility, which usually takes years to develop, so regular check-ups with a dental practitioner are needed for early identification and management of periodontitis. Furthermore, periodontitis is associated with diabetes, cardiovascular disease, rheumatoid arthritis and Alzheimer’s disease [24]. It is therefore recommended SROH be included in check-ups with healthcare professionals and a collaborative approach be used to manage physical health conditions and dental care in rural areas of Australia.

Despite the high prevalence of oral conditions, people generally self-rated the health of their teeth and gums as good (41.8%) or very good (28.0%). This perception is consistent with research comparing the perceptions of oral health from national health surveys in Australia, Canada, New Zealand and the United States [25]. Although the levels of dental disease in Australia were higher than elsewhere, Australians presented the most positive self-rated oral health [25]. An explanation for the differences between clinical measures and self-rated oral health in the Australian population could be that self-rated health is sign- and symptom-based (pain or no pain) and related to function (if the number of teeth or a well-adapted denture allows for eating well). In the present study, participants with filled teeth did not have a negative SROH (there was no significant difference), which could be due to the fact that a filled tooth allows functionality and is not associated with symptoms. This highlights the importance of having a broader perspective of the determinants of an individual’s oral health status, taking clinical and self-rated measures into account [6].

Previous studies have reported that self-rated oral health is valid and reliable for assessing oral health status [8,21,26,27]. This measure has been used to evaluate oral health status in epidemiological studies, indicating a similar trend to clinical data, particularly for those representing dental caries experience, tooth loss and need for a dental prosthesis [7,8,21,26,27]. However, being a subjective measure, differences in sensitivity and specificity have been recognised between different cultures and populations (rural versus urban). These differences may be influenced by socioeconomic status, health literacy and access to dental care [8,10,26].

The findings from this study cannot be directly compared to results from the National Study of Adult Oral Health (NSAOH) [11] due to methodological differences (e.g., the criteria used to assess dental caries history). However, this study provides insight into the state of oral health in a rural area of Victoria and the high prevalence of oral pathologies, which needs further exploration in other rural regions of Australia. An association between self-rated oral health and oral health clinical parameters suggests that individuals are aware of the impact of oral disease on oral health status.

Considering oral epidemiological research in rural areas is scarce, this study’s results provide new knowledge on oral health and how the population values their oral health status in rural areas of Victoria. This provides valuable information from a clinical point of view and for planning and decision making in oral healthcare. According to this study, SROH measures are valid in reflecting an individual’s oral health status in terms of experiencing tooth decay, missing teeth and having clinical attachment loss. The findings of this study suggest that self-rated oral health can be used as a proxy measure for oral health status in the Australian rural population and provides insight into the clinical care needs of individuals, allowing those who require dental care to be prioritised.

## 5. Conclusions

The results of the present study suggest that SROH measures are valid in assessing the oral health status of the rural Australian population. Most participants who reported their oral health as poor or fair had clinical care needs. Poorer SROH was also associated with less frequent dental visits and lower self-rated general health. The results emphasise the significance of integrating SROH techniques into healthcare appointments to help identify those requiring dental care in rural Australian areas where the dental care workforce is in short supply.

## Figures and Tables

**Table 1:** Sociodemographic characteristics of the participants.

Variables	Categories	*n* (%)
Age	<30	34 (6.0)
	30–39	66 (11.6)
	40–49	53 (9.3)
	50–59	111 (19.5)
	60–69	137 (24.0)
	70 and over	169 (29.6)
Sex	Male	256 (44.7)
	Female	317 (55.3)
Education	Some secondary	193 (36.8)
	Secondary complete	58 (11.1)
	Trades	120 (22.9)
	Tertiary	153 (29.2)
Location	Benalla/Cobram/Seymour	295 (51.4)
	Shepparton/Mooroopna	279 (48.6)
Self-rated general health	Excellent	26 (4.5)
	Very good	94 (16.4)
	Good	184 (32.1)
	Fair	190 (33.1)
	Poor	80 (13.9)
Self-rated oral health	Excellent	29 (5.3)
	Very good	152 (28.0)
	Good	227 (41.8)
	Fair	78 (14.4)
	Poor	57 (10.5)
Time since last dental visit	12 months or less	280 (50.9)
	Between 1 and 2 years	107 (19.5)
	Longer than 2 years	163 (29.6)

**Table 2:** Univariate associations between sociodemographic characteristics, clinical oral health variables and self-rated oral health.

Self-Rated Oral Health (%)				
Variables	*n*	Negative	Positive	Odds Ratio (95% CI ‡)
Age (years)	574	57.6 (16.3) †	58.2 (16.2)	N.S.
Sex				N.S.
Male	241	27.0	73.0	
Female	307	24.4	75.6	
Level of education				N.S.
Some secondary	183	32.2	67.8	
Secondary complete	54	31.5	68.5	
Trades	117	20.5	79.5	
Tertiary	149	23.5	76.5	
Locality of residence				N.S.
Shepparton/Mooroopna	268	24.3	75.7	
Shire capitals	281	27.0	73.0	
Self-rated general health ***				3.58 (2.24–5.71)
Negative	92	48.9	51.1	
Positive	455	21.1	78.9	
Time since last dental visit				
12 months or less **	275	21.1	78.9	0.63 (0.42–0.94)
Between 1 and 2 years	102	19.6	80.4	N.S.
Longer than 2 years ***	151	36.4	63.6	2.19 (1.45–3.32)
Dentition				
Edentulous (no natural teeth)	37	21.6	78.4	N.S.
Inadequate dentition ***	136	39.0	61.0	2.35 (1.55–3.58)
Adequate dentition ***	376	21.3	78.7	0.49 (0.33–0.73)
Dentures				N.S.
No dentures	380	23.4	76.6	
Partial denture	133	33.1	66.9	
Complete denture	36	22.2	77.8	
DMFT	549			
Decayed teeth § ***		2.2 (2.7) †	1.0 (1.5)	1.38 (1.24–1.53)
Filled teeth §		5.9 (5.0) †	6.8 (5.2)	N.S.
Missing teeth **		12.3 (8.8) †	9.9 (9.1)	1.04 (1.02–1.06)
CPI				
Gingival bleeding:				
Presence	403	27.5	72.5	N.S.
Absence	171	21.2	78.8	
Periodontal Pocket:				
0–3 mm	366	21.6	78.4	0.50 (0.33–0.76)
4–5 mm	113	29.7	70.3	N.S.
≥6 mm	45	50.0	50.0	3.24 (1.73–6.08)
Toothache ***				1.66 (1.24–2.23)
Yes	47	51.1	48.9	
No	417	18.5	81.5	
Frequency of toothache				
Never ***	253	15.4	84.6	0.34 (0.22–0.52)
Hardly ever	148	23.6	76.4	N.S.
Occasional ***	115	40.0	60.0	2.38 (1.53–3.69)
Fairly often ***	29	69.0	31.0	7.33 (3.25–16.53)
Clinical attachment loss § ***				
0–3 mm	262	19.1	80.9	0.50 (0.34–0.75)
4–5 mm	187	29.4	70.6	N.S.
≥6 mm	100	36.0	64.0	1.84 (1.16–2.92)

† Mean and standard deviation; ‡: CI = confidence interval; §: dentate participants only. * *p* < 0.05; ** *p* < 0.01; *** *p* < 0.001. Figures may not add up to 100% due to missing values.

**Table 3:** Regression coefficient and odds ratios for the oral clinical variables predicting negative self-rated oral health.

Variables in Equation	Β-Coefficient	*p*-Value	Odds Ratio	95% Confidence Interval
Time since last dental visit (12 months or less = ref)		0.03		
Between 1 and 2 years	0.03	0.99	1.00	0.47–2.10
Longer than 2 years	0.76	<0.01	2.14	1.17–3.93
Self-reported general health (Positive = ref)	0.84	<0.01	2.32	1.26–4.29
Number of Missing teeth	0.04	<0.01	1.05	1.01–1.08
Number of teeth with dental caries	0.24	<0.01	1.28	1.11–1.46
Clinical attachment loss (0–3 mm = ref)		0.01		
4–5 mm	0.64	0.04	1.90	1.02–3.54
≥6 mm	0.97	<0.01	2.63	1.29–5.38
Toothache (No = ref)	0.84	0.06	2.33	0.97–5.56
Frequency of toothache (Never/hardly ever = ref)	0.94	<0.01	2.56	1.35–4.84
Constant	−3.30			

The variance in self-rated oral health accounted for using the final model was 27.7% (η^2^ = 0.277).

## Data Availability

Data can be made available on reasonable request using an application form from the Crossroads Chief Investigator (D.S.).

## Appendix A

**Table A1:** Self-rated oral health questions extracted and adapted from Crossroads II study.

Question	Answers
Overall, how would you rate the health of your teeth and gums?	(1) Excellent (2) Very good (3) Good (4) Fair (5) Poor
Do you have a decayed tooth?	(1) Yes (2) No
Do you have bleeding gums?	(1) Yes (2) No
Do you have loose teeth?	(1) Yes (2) No
Do you have toothache and/or mouth pain?	(1) Yes (2) No
In the last 12 months, how often have you had painful aching in your mouth?	(1) Never (2) Hardly ever (3) Occasional (4) Fairly often
